# Measurement of muscle length-related electromyography activity of the hip flexor muscles to determine individual muscle contributions to the hip flexion torque

**DOI:** 10.1186/2193-1801-3-624

**Published:** 2014-10-22

**Authors:** Takumi Jiroumaru, Toshiyuki Kurihara, Tadao Isaka

**Affiliations:** Graduate School of Sport and Health Science, Ritsumeikan University, 1-1-1 Noji Higashi, Kusatsu, Shiga, 525-8577 Japan; Shiga School of Medical Technology, 967 Kitasaka-cho, Higashiomi, Shiga, 527-0145 Japan; Department of Sport and Health Science, Ritsumeikan University, 1-1-1 Noji Higashi, Kusatsu, Shiga, 525-8577 Japan

**Keywords:** Isometric hip flexion, Surface electromyography, Muscle length-joint angle relationship, Synergistic muscles

## Abstract

This study aimed to investigate muscle length-related electromyography (EMG) of the iliopsoas (IL) and other hip flexor muscles to determine individual muscle contributions to the hip flexion torque. Ten healthy sedentary young men participated in the EMG experiment. A subgroup of six subjects underwent a magnetic resonance imaging (MRI) measurement to confirm the region of the skin over the IL. Surface EMG signals were sampled from the IL, rectus femoris (RF), sartorius (SA), and tensor fasciae latae (TFL) using an active electrode. The subjects performed maximum voluntary isometric hip flexion with the right hip joint set at -10°, 0°, 30°, and 60°. The root mean square (RMS) value for the TFL at 30° (0.81 ± 0.19, p <0.005) and 60° (0.66 ± 0.17, p <0.001) and the SA at 60° (0.62 ± 0.24, p <0.005) were significantly decreased compared with those at 0°. However, the RMS value for the IL and RF did not change significantly. The RMS value and muscle length changes were significantly correlated in the IL (r =0.39, p <0.05), SA (r =0.51, p <0.001), and TFL (r =0.70, p <0.001), but not in the RF (r =0.22, p =0.180). We conclude that, in a hip joint flexed position, the contribution of the IL to hip flexion movement is relatively larger than that of the other hip flexor muscles.

## Background

During actual body motion involved in daily activities and sports, muscles often exert force at appropriate joint angles. Therefore, it is important to examine the characteristics of muscle force production associated with the changing joint angle. By changing the joint angle, the muscle length changes, but the way that the muscle length changes varies within each muscle. Therefore, the difference in muscle length changes affects the electromyography (EMG) amplitude. Namely, there are muscles that change their muscle length in response to changing joint angles and those that do not. Muscles that are not easily influenced by changing joint angles exhibit an increased relative contribution to force production as a result of the changing joint angles. The force production of each muscle was estimated from quantum analysis of EMG (Maffiuletti and Lepers 2003; Pincivero et al. 2004; Ruiter et al. 2008; Watanabe and Akima 2011; Saito et al. 2013), based on the existence of a strong relationship between contractile force and isometric contractions on EMG (Dolan et al. 2001; Stokes 2005).

Previous studies have shown that even for synergist muscles, which perform the same functions as agonist muscles, the activity of each muscle changes along with joint angle (Pincivero et al. 2004; Watanabe and Akima 2011). Studying the relative activation of individual synergists to determine the contribution of each muscle to the force production is very important to the development of training programs and targeted physical therapy, and many previous studies have used the amplitude of EMG for this purpose (Maffiuletti and Lepers 2003; Ruiter et al. 2008). The relationship between joint angle and EMG activity is essentially derived from muscle length-related changes. Muscle length is well known to change along with joint angle, and these changes affect muscle force. Consequently, individual muscle contributions to the hip flexion torque can be determined by calculating the muscle length-EMG relationship.

The contribution of muscles that show recordable responses with surface EMG (sEMG) to changing joint angle during submaximal exercise has been reported in quadriceps (Watanabe and Akima 2011; Saito et al. 2013); however, such contribution has not yet been revealed for the hip flexor muscles. Since the iliopsoas (IL) muscle group, which consists of the psoas major and iliacus muscles, is located in the deepest region of the trunk, the activity of the IL has seemed unrecordable with sEMG. The IL is considered the primary muscle for hip flexion because of the larger physiological cross-sectional area of the IL compared to other hip flexor muscles (Hoy et al. 1990; Juker et al. 1998). Additionally, the cross-sectional area of the IL has demonstrated significant correlations with the walking ability of elderly and running ability of athletes (Kim et al. 2000, 2001; Copaver et al. 2012). Few studies have investigated the IL activity using fine-wire electrodes (Andersson et al. 1995, 1997; Juker et al. 1998; Park et al. 2013), yet the research was never applied to the relative contribution of the IL to hip flexion torque.

The purpose of this study was to investigate the relative contribution of hip flexor muscles by testing the relationship between muscle length and EMG activity of the hip flexors, including the IL. In recent years, we have established a method for recording sEMG signals from the IL (Jiroumaru et al. 2014), which has made it possible to study the contribution of individual hip flexor muscles. However, our previous study only confirmed the effectiveness of this method for isometric contractions at 0° of hip flexion. Therefore, we used magnetic resonance imaging (MRI) to confirm the region of the skin over the IL at different hip joint angles, thus providing evidence that this method can record accurate EMG signals at different hip joint angles.

## Results

**Figure 1:**
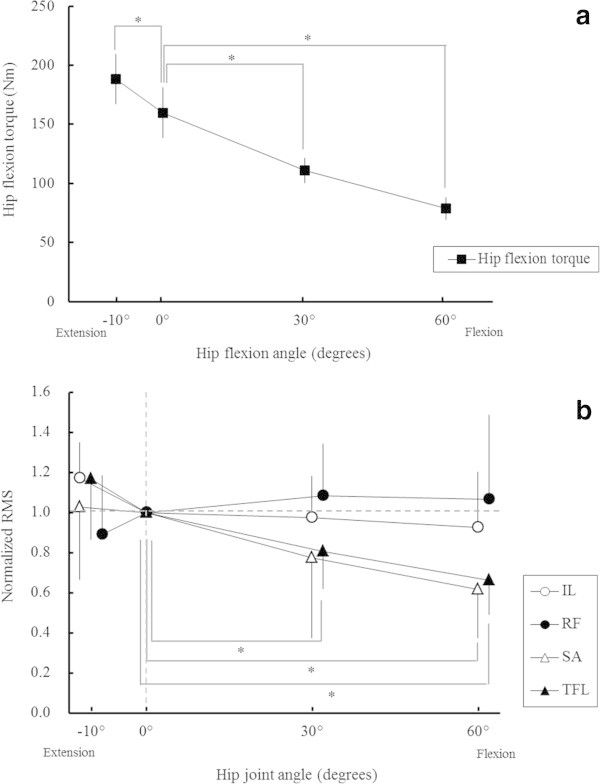
**Hip flexion torque and electromyography during isometric contraction. (a)** Maximum voluntary hip flexion torque during isometric contraction at four hip joint angles. Values are presented as mean ± standard deviation. *p <0.05 vs. 0° **(b)** Normalised electromyographic activity at four hip joint angles. The average root mean square (RMS) values for maximum voluntary contraction at four hip joint angles were normalised by the RMS value at a hip joint angle of 0°. Values are presented as mean ± standard deviation. *p <0.05 vs. 0° IL, iliopsoas; RF, rectus femoris; SA, sartorius; TFL, tensor fasciae latae.

Hip flexion torque at -10° (118.4% ±6.5%), 30° (70.1% ±4.6%), and 60° (50.2% ±8.5%) was significantly different from that at 0° (159.7 ± 21.3 Nm) (p <0.001 for each case) (Figure 1a).

A significant hip joint angle interaction in normalised RMS values was found (p <0.05) (Figure 1b). The normalised RMS value for the TFL at 30° (0.81 ± 0.19, p <0.05) and 60° (0.66 ± 0.17, p <0.001) and for the SA at 60° (0.62 ± 0.24, p <0.005) were significantly decreased compared with that at 0°. On the other hand, the normalised RMS value for the IL and RF were not significantly changed within the four hip joint angles (Figure 1b).

**Figure 2:**
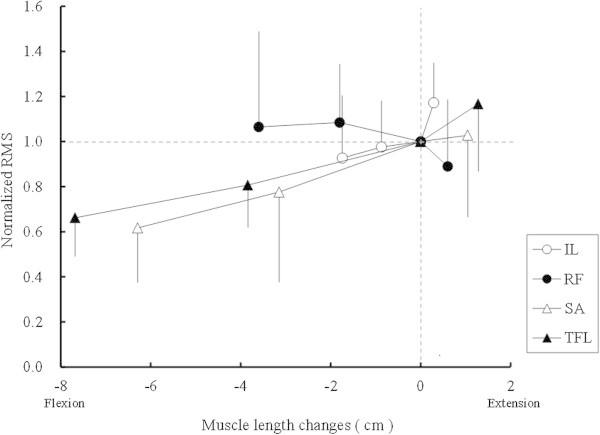
**Relationship between normalised electromyographic activity and muscle length changes.** Average root mean square (RMS) values for maximum voluntary contraction at four hip joint angles were normalised by the RMS value at a hip joint angle of 0°. Values are presented as means and standard deviations. IL, iliopsoas; RF, rectus femoris; SA, sartorius; TFL, tensor fasciae latae.

There were significant correlations between the RMS value and the muscle length changes in the IL (r =0.39, p <0.05), SA (r =0.51, p <0.001), and TFL (r =0.70, p <0.001), but not in the RF (r =0.22, p =0.180) (Figure 2). Analysis of covariance (ANCOVA) revealed that the slopes and intercepts of the regression lines for the IL, SA, and TFL were not significantly different.

The cross-correlation values within each muscle at four different joint angles are shown in Table 1. Average peak R_xy_ values between SA, RF, TFL, and IL within the range of -10° to 60° were less than 0.2, except for that of IL with RF at 60° (0.221 ± 0.112) (Table 1).

**Table 1:** **Mean peak cross-correlation (R**_**xy**_ **) with standard deviation in parentheses for each muscle electrode**

	-10°	0°	30°	60°
IL vs. SA	0.157 (0.188)	0.187 (0.087)	0.183 (0.085)	0.142 (0.090)
IL vs. RF	0.153 (0.072)	0.160 (0.077)	0.178 (0.068)	0.221 (0.112)
IL vs. TFL	0.092 (0.030)	0.104 (0.044)	0.089 (0.033)	0.095 (0.030)
SA vs. RF	0.167 (0.048)	0.133 (0.052)	0.151 (0.051)	0.171 (0.064)
SA vs. TFL	0.073 (0.070)	0.087 (0.023)	0.096 (0.039)	0.096 (0.025)
RF vs. TFL	0.078 (0.017)	0.079 (0.026)	0.083 (0.034)	0.092 (0.035)

IL, iliopsoas; RF, rectus femoris; SA, sartorius; TFL, tensor fasciae latae.

Perimeter IL on the marker position (electrode location for the IL) was more than 10 mm at all hip joint angles (Table 2). We observed a significant difference in the length of perimeter IL between a hip joint angle of 60° and the other three hip joint angles (all p <0.05) (Table 2). Moreover, the mean area of the superficial region of the IL was significantly different between 60° and all the other angles (Table 2). The depth from the surface to the IL was below 10 mm at -10° and 0° (Table 2). We observed a significant difference in the depth from the surface to the IL between a hip joint angle of 60° and the other three hip joint angles (all, p <0.05) (Table 2). At 0°, the values in the supine position were approximately 3 mm smaller than values in the lateral position (Table 2).

**Table 2:** **The perimeter IL, area of the superficial region, and depth from the surface to IL**

	-10°	0°	30°	60°
Perimeter IL (1 cm proximal from the marker position), mm	26.0 (9.4)*	20.9 (7.6)*	13.6 (2.0)*	7.0 (4.9)
Perimeter IL (marker position), mm	25.1 (5.7)*	23.6 (3.7)*	19.8 (2.7)*	10.2 (5.7)
Perimeter IL (1 cm distal from the marker position), mm	21.1 (5.2)*	17.7 (3.9)*	14.6 (2.3)	5.2 (3.9)
Area of the superficial region of the IL, mm^2^	1,192.2 (394.2)*	1,018.7 (312.1)*	642.2 (143.4)*	290.4 (155.0)
Depth from the surface to the IL in the lateral positions, mm	7.9 (4.1)*	9.6 (4.1)*	11.4 (4.5)*	20.9 (10.5)
Depth from the surface to the IL in the supine positions, mm		6.5 (4.1)		

^*^p <0.05 vs. 60°.

IL, iliopsoas.

Standard deviations are noted in parentheses.

## Discussion

The objective of this study was to investigate the relative contribution of hip flexor muscles including the IL, by testing the relationship between muscle length and EMG activity of the hip flexors. The main finding of this study was that hip flexor EMG activity differed according to change in the length of each muscle. It is necessary to determine the muscle length-EMG relationship of each muscle to elucidate the degree of contribution, because muscle length changes muscle force through a force-length relationship (Gordon et al. 1966; Rassier et al. 1999). Thus far, previous studies have only reported the relationship between joint angle and EMG activity, and not the relationship between muscle length and EMG activity. To the best of our knowledge, this is the first study to use the sEMG technique to investigate the relationship between muscle length and EMG activity of the hip flexors.

The peak torque was obtained at -10° of hip flexion, and there was a significant decrease in torque as the angle of hip flexion increased. This reduction is generally in agreement with previous studies (Williams and Stutzman 1959; Kulig et al. 1984). A significant RMS reduction in the TFL and SA with increasing hip flexion angle implies that the contributions of the TFL and SA decreased when exerted in the flexed position. Therefore, with the hip joint in flexed position, the contribution of the IL to hip flexion movement is relatively larger than that of the other hip flexor muscles. The change in degree of contribution according to joint angle has been shown in many previous studies for quadriceps femoris muscles (Pincivero et al. 2004; Ruiter et al. 2008; Watanabe and Akima 2011; Saito et al. 2013). Even if maximum activation of each muscle is achieved, maximum joint torque is altered by moment arm length and change in the length of each muscle, which are affected by joint angle (Lieber and Butler 1992).

In the present study, there were significant correlations between muscle length and EMG activity of the IL (r =0.39, p =0.03), TFL (r =0.70, p =0.001), and SA (r =0.51, p =0.001). In contrast, the RMS value for the RF was not correlated with muscle length (r =0.22, p =0.180) (Figure 2). When plotting the relationship between changes in muscle length and normalised RMS values, no significant difference was identified in the slope of the regression lines for the IL, SA, or TFL. The previous study by Hawkins and Hull (1990) indicated that muscle length of the IL scarcely changes with hip joint angle, despite the TFL and SA lengths changing extensively. It is supposed that EMG activity of the IL in a hip joint flexed position is relatively constant due to less change in muscle length, whereas that of the TFL and SA are influenced by the vast change in muscle length with various flexion angles. Among the hip flexor muscles, the RF has the shortest muscle fibre length (Hoy et al. 1990; Ward et al. 2009). Difference in muscle fibre length causes differences in both whole muscle length and the width of the force-length curve (Lieber and Butler 1992), which may be the reason for the nonsignificant correlation between muscle length and EMG activity. The width of the force-length curve differs across muscles and individuals (Herzog and ter Keurs 1988; Lieber and Butler 1992; Winter and Challis 2010); therefore, optimal length values may also differ.

Previous studies indicated the possibility that the muscle activity of the iliacus and the psoas major differ in various human movements (Andersson et al. 1995, 1997). sEMG from IL in this study cannot distinguish the activity of the iliacus and psoas major; therefore, it is necessary in future studies to clarify which of the two muscle activities is reflected to a greater degree in the superficial region of the IL. However, the iliacus and psoas major demonstrated similar levels of muscle activation during isometric hip flexion at different joint angles (Andersson et al. 1995), although activation of the psoas major was observed for lateral trunk stabilization, when the iliacus was silent (Andersson et al. 1995). In addition, the activation of iliacus and psoas major during walking was almost equivalent (Andersson et al. 1997). This previous study indicated that the timing of onset of each muscle during walking was different; i.e. the iliacus was engaged prior to the psoas major at the latter half of the swing phase, but the magnitude of activation was the same.

The method for recording sEMG signals in our previous paper (Jiroumaru et al. 2014) must be carefully applied, because, in that study, we only measured sEMG at 0° of hip flexion. The electrodes used in the present study were 5 × 5 mm in size, with an interelectrode distance of 10 mm; thus, an area of ≥100 mm^2^ for the superficial region is required to attach the surface electrode sensors. We determined that the superficial region of the IL was sufficient at hip joint angles of -10°, 0°, 30°, or 60° (Table 2). Theoretically, the 10-mm interelectrode distance used in the present study can detect 10 mm of muscle activity below the skin. Therefore, we determined the depth from the surface to the IL was sufficient at -10° and 0°. Besides, after comparing values in the lateral and supine positions at 0°, the value in the supine was approximately 3 mm smaller than the value in the lateral position. The depth at 30° was 11.4 ± 4.5 cm. As a result, we can predict that the depth from the surface to the IL will also be below 10 mm at 30° in the supine position. Furthermore, considering the cross-correlation analysis, the levelling off of existent crosstalk was 0.2 in a previous study (Winter et al. 1994). Most Rxy values in this study were below that threshold. The most plausible crosstalk signal of the IL from adjacent muscle was RF at 60° (0.221 ± 0.112), and no significant crosstalk signals were determined between the sartorius (SA), rectus femoris (RF), tensor fasciae latae (TFL), and IL within the range from -10° to 30° (Table 1). A previous study indicated that the thickness of subcutaneous fat increases EMG crosstalk at nearby surface recording sites (Kuiken et al. 2003). There was substantial superficial fat over the IL at 60° (Table 2). Hence, we considered that recording the IL EMG activity at 60° is impractical, while the crosstalk of the adjacent muscles can be ignored within the range from -10° to 30°. Consequently, combined with these results of cross-correlation, the findings indicated that the EMG signals from the IL at 30° can be recorded with little impact from the other muscles. During normal walking, the rotational angle range of hip flexion/extension was between -10° and 25° (Kuster et al. 1995; Kerrigan et al. 1998). Therefore, recording the IL EMG activity during normal walking is possible using a surface electrode on the IL. sEMG activity of the hip flexors, including the IL, during walking may provide a more detailed level of understanding of IL function, which is related to walking ability.

The limitations of this study were as follows. First, the experimental protocol only involved static contraction. The relative displacement of skin and muscle during dynamic movement was ignored; therefore, the application of this method to dynamic movement requires careful examination. Further study is needed to elucidate the relative displacement effect during normal human movement. Second, the equation used in this study for estimating the muscle length changes in the IL is from the pelvis, which is the originating part of the iliacus and does not include the lumbar spine from which the psoas major originates. Therefore, the estimation equation does not yield the optimal evaluation results for the muscle activity of the IL, which reflects the muscle activities of both the iliacus and psoas major. It is necessary to design an equation to estimate muscle activity changes of the iliacus and the psoas major separately. However, the change in hip joint flexion/extension rotational angle induces only limited changes to the lengths of the iliacus and psoas major, although the iliacus and the psoas major have a different origin (Hoy et al. 1990). Thirdly, the additional MRI measurements of this study were obtained from muscles in the passive state. Earlier studies have reported that muscles in isometric contractions undergo relative anatomic changes (Hodgson et al. 2006). The innervation zone changes with the joint angle (Martin and MacIsaac 2006) and/or submaximal isometric contractions (Piitulainen et al. 2009). Thus, these changes may affect the amplitude of the EMG. The relative anatomical changes of muscles and innervation zone during isometric contractions should be included in future research. Finally, the hip flexion angle in the range of -10° to 30° was considered in this study, but the internal/external rotation of the hip joint and adduction/abduction movement of the pelvic were neglected. This present assessment is adequate for normal walking; however, during other movements such as fast walking or running, or when turning to change direction, greater internal/external rotation movements of the hip joint or adduction/abduction movements of the pelvic will occur.

## Conclusion

In conclusion, this study evaluated the relationship between muscle length and EMG activity of the hip flexor muscles, including the IL, during maximum voluntary isometric contraction. As the angle of hip flexion increased, muscle length of the TFL and SA changed significantly and EMG activity decreased. Conversely, muscle length of the IL and RF scarcely changed and there was little change in EMG activity. These results suggest that the degree of contribution from the IL and RF increases as the angle of hip flexion increases. This finding may be useful for the evaluation of hip flexor muscle strength and in designing rehabilitation and training for physical therapists or athletic trainers.

## Methods

### General study design

During maximum voluntary isometric contraction (MVIC) of hip flexion at four different hip joint angles, sEMG signals from hip flexor muscles were recorded. Muscle length was calculated with limb length and the hip joint angle according to the equation by Hawkins and Hull (1990) and was converted to the relationship between the muscle length and EMG activity of each muscle. As an additional imaging study in only six subjects, we also confirmed the position and size of the superficial region of the IL based on changes in the hip joint angle measured on MRIs.

### Subjects

Ten healthy sedentary young men (age: 27.2 ± 2.7 years, weight: 67.2 ± 6.3 kg, height: 172.0 ± 3.8 cm), with no orthopaedic abnormalities of the trunk or hip muscles, voluntarily participated in the sEMG experiment. A subgroup of six subjects underwent further MRI measurements (age: 28.7 ± 1.8 years, weight: 69.3 ± 7.1 kg, height: 171.2 ± 3.9 cm). Before the experiments, the procedure, purposes, and risks associated with the study were explained to the subjects, and written informed consent was obtained from all subjects. The study protocol was approved by the Ethics Review Board of Ritsumeikan University Biwako-Kusatsu Campus (IRB-BKC-2012-06), and all experimental procedures were performed in accordance with the Declaration of Helsinki.

### Hip flexion task

**Figure 3:**
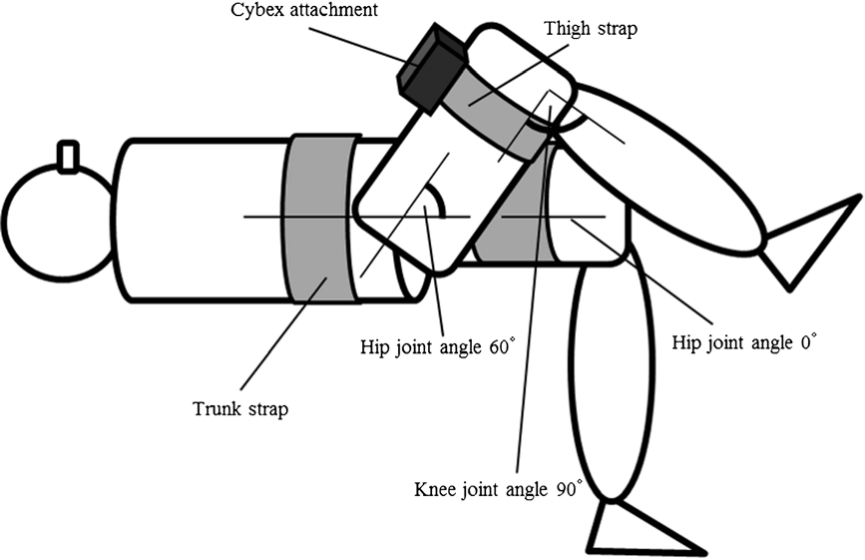
**Schematic diagram of hip flexion task.** During hip flexion tasks, the trunk and left thigh were fixed to the bed by straps, with the knee joints flexed to 90° and the right hip joint angle set at -10°, 0°, 30°, and 60°. The right thigh was attached to a dynamometer.

Isometric hip flexion torque was measured by a commercially available dynamometer (CYBEX 770; Lumex Inc., Bay Shore, NY, USA). All subjects participated in trial sessions of the MVIC task at least 1 wk before the experiment for familiarisation. For the task, the subject lay supine on a bed, with the trunk and left thigh fixed by a strap. The right hip joint was randomly set at -10°, 0°, 30°, and 60°, with the knee joints flexed to 90° (Figure 3). The subject then performed MVIC twice for each joint angle, with a 2-min interval between trials. During contraction, the subject was asked to exert force by only hip flexion action. If two exerted forces at a hip joint angle differed by more than 5% between trials, an additional trial was imposed. The MVIC test involved a force-increasing phase (1–2 s), sustained maximum phase (2 s), and relaxation phase. For each contraction, the force value was sampled and averaged across 2 s during the sustained phase. Then the two peak forces were averaged, and the average value was used as the representative MVIC value for each hip joint angle.

### Surface electromyography recording

The details of the apparatus and procedures have been described previously (Jiroumaru et al. 2014). Briefly, sEMG signals were collected from four agonist hip flexor muscles (Oatis and Kligler 2009): the rectus femoris (RF), sartorius (SA), tensor fasciae latae (TFL), and IL. An active electrode with a 5 × 5-mm diameter was used, and an inter-electrode distance was set at 10 mm (MQ8/16 16-bit EMG amplifier; Kissei Comtec, Nagano, Japan). An active electrode aims to measure accurate biosignals by creating higher impedance than skin impedance of the measurement points. For the matter, a manufacturer guarantees that the method enables the investigation to measure dynamic actions compared to the conventional researches; in addition, noises are rarely included in the signals. This is because; it is structured to digitalize signals nearby an electrode. At the same time, the manufacturer also guarantees a reduction of the process time which is necessary for pre-procedure. EMG signals were recorded at a sampling frequency of 1,000 Hz (16-bit) by the telemetric system (MQ16; Kissei Comtec), and data were collected and processed using analysis software (Kine Analyzer; Kissei Comtec).

**Figure 4:**
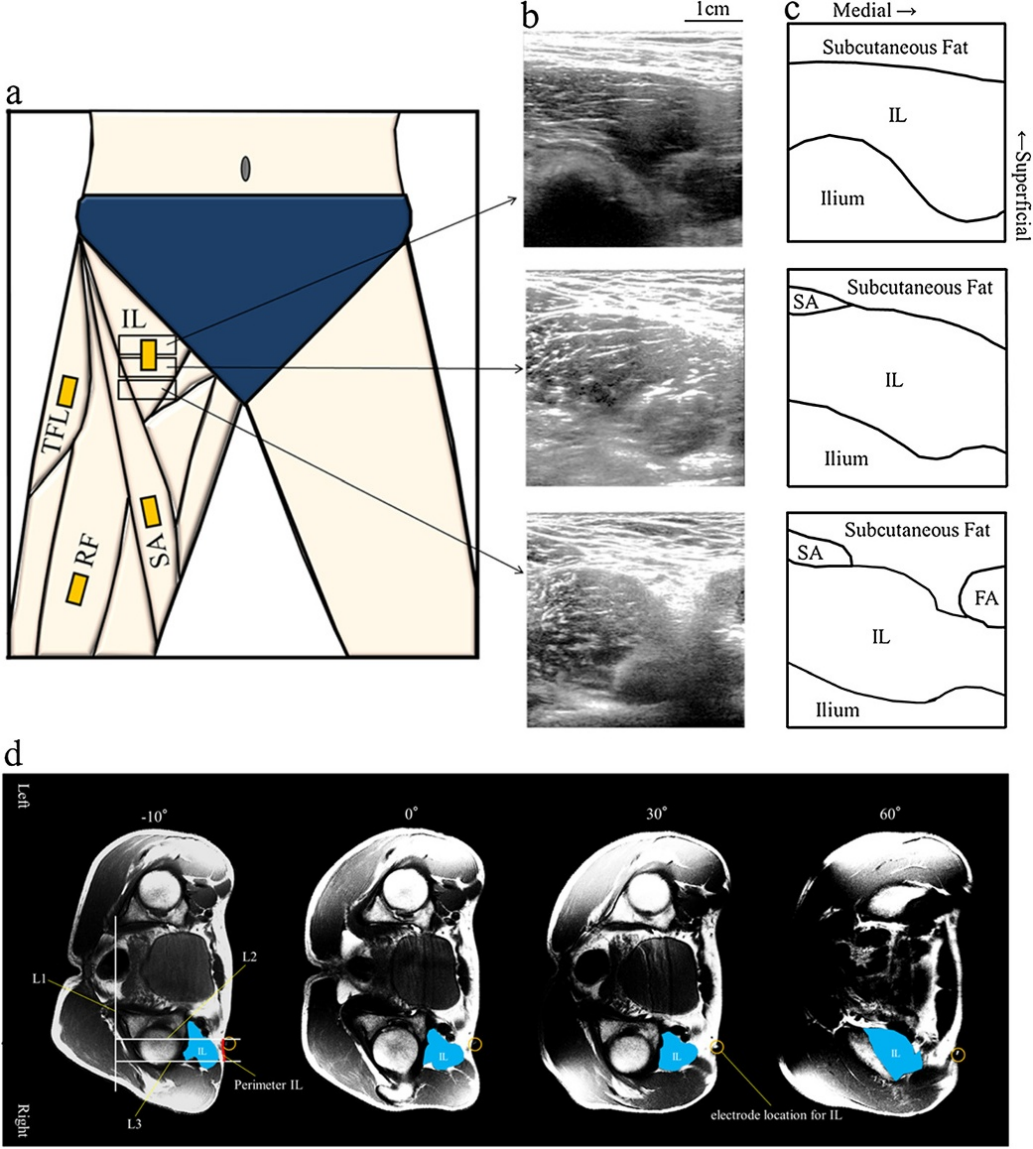
**Confirmation of the electrode positions. (a)** Electrode positions on the hip flexor muscles. Representative maximal perimeter IL, 1 cm distal, and 1 cm proximal axial ultrasonographic images **(b)** and schematic illustrations **(c)** of the superficial region of the IL from under the groin at a hip joint angle of 0°. Representative axial magnetic resonance images with recumbent posture on the right side of the examination bed with the right hip joint set at -10°, 0°, 30°, and 60° **(d)**. The electrode location on the IL is surrounded by a circle. L1, a straight line drawn along the posterior end line of the ilium on both sides; L2 and L3, two lines drawn vertically to L1 at the medial and lateral edges of the IL (Jiroumaru et al. 2014). FA, femoral artery; IL, iliopsoas; RF, rectus femoris; SA, sartorius; TFL, tensor fasciae latae.

The electrode for the RF was placed at the midpoint between the anterior inferior iliac spine (ASIS) and the superior border of the patella. The electrode for the SA was placed 8 cm distal from the ASIS along the line between the ASIS and the median of the tibial tuberosity parallel to the estimated muscle fibres. The electrode for the TFL was placed at the midpoint between the ASIS and the head of the greater trochanter. The electrode for the IL was placed at a level 3–5 cm distal from the ASIS (Jiroumaru et al. 2014), while the ultrasound probe was applied directly under the groin to identify the subcuticular existence of the IL. These electrodes were placed parallel to the longitudinal axis of the muscle (Figure 4). The reference electrode was attached on the right patella. Prior to attaching the electrodes, the skin was shaved, abraded, and cleaned with alcohol.

To calculate the root mean square (RMS) value for the EMG signal during each contraction, the EMG signal was sampled across 2 s of the sustained phase, as was the torque. The two RMS values during MVIC were averaged for each hip joint angle. Average RMS values for MVIC at the four hip joint angles were normalised by the RMS value at a hip joint angle of 0°. In order to verify that there was no effect of crosstalk interference between the muscles, we conducted cross-correlation analysis within each signal. The 2-s recording signals for each muscle were cross-correlated with each other according to the following formula presented by Winter et al. (1994):


where *R*_*xy*_*(τ)* is the cross-correlation function, *x*(*t*) and *y*(*t*) are any two myoelectric signals, *T* is the length of the records being correlated, and τ is the temporal phase shift between the two signals.

### Calculation of muscle length

Along with hip flexion angle, we calculated the change in muscle length of the IL, RF, SA, and TFL by using the following previously reported estimation equations (Hawkins and Hull 1990):


where *ΔL* is the change in muscle length based on anatomic limb position, and *α* and *β* are the hip and knee joint angles in degrees, respectively. Thigh length was defined as the length from the greater trochanter to the lateral epicondyle of the femur.

### Magnetic resonance imaging measurement

The subgroup of six subjects underwent MRI measurement on the day after the sEMG recording. The subject lay relaxed recumbent on his right side on the examination bed, with the right knee flexed to 90° and the right hip joint set at -10°, 0°, 30°, and 60°. The pelvis was immobilized by the straps. And the pads and cushions were placed beside the subjects to restrict the trunk movement. We attached a water-soluble marker to the skin at the placement of the IL electrode. Using a 1.5-T MRI system (Signa HDxt; GE Healthcare UK Ltd., Buckinghamshire, UK), the parameters were set as follows: spin-echo; repetition time, 400 ms; echo time, 7.8 ms; matrix, 512 × 512; field of view, 400 × 400 mm; no gap; slice thickness, 10 mm; and number of excitations, 2. Consecutive axial images were obtained along the ASIS to the lesser trochanter of the femur. From these images, the perimeter of the skin facing the IL was measured (perimeter IL) (Figure 4d). In addition, we measured the depth from the surface to the IL. Because of the configuration of the MRI system used in the present study, we only measured lateral positions at -10°, 30°, and 60° and also in the supine at 0°. The detailed measurement procedure has been previously described (Jiroumaru et al. 2014). Briefly, perimeter IL was defined as the length of the medial and lateral edges of the superficial region of the IL. The proximal-distal length facing the IL was defined as the number of the images with perimeter IL ≥10 mm. We also estimated the area of superficial region of the IL using trapezoidal approximation. Considering the electrode size of 5 × 5 mm and inter-electrode distance of 10 mm, an area ≥100 mm^2^ would be needed to attach a surface electrode sensor on the muscle of interest.

### Statistical analysis

All data are provided as mean ± standard deviation. The MVIC, perimeter IL, area of the superficial region of the IL, and depth from the surface to the IL were analysed using one-way (hip joint angle) analysis of variance (ANOVA). When a significant difference among joint angles was apparent, Dunnett’s test of MVIC was applied as a post-hoc test to compare all other data points to values at a hip joint angle of 0°. Dunnett’s test of perimeter IL and the area of the superficial region of the IL were applied as a post-hoc test to compare all other data points to values at a hip joint angle of 60°. Additionally, Dunnett’s test of depth from the surface to the IL was applied as a post-hoc test to compare all other data points to values at a hip joint angle of 60°. Normalised RMS values during MVIC were analysed using two-way (muscle × hip joint angle) ANOVA. In the case of a two-factor interaction or a main effect for hip joint angle, Dunnett’s test was applied as a post-hoc test to compare all other data points to values at a hip joint angle of 0°. Pearson's correlation coefficient analysis was used to assess the relationship between RMS value and muscle length of each muscle. Then the slopes and intercepts of the regression lines among the individual muscles were estimated using ANCOVA. The peak R_xy_ value of a cross-correlation function was calculated as a correlation-based index of crosstalk from each muscle. In accordance with Winter et al. (1994), cross-correlation values over a 2-s period without a common signal were confirmed as 0.2, and the value of 0.2 was set at the levelling off threshold. Statistical analyses were performed using SPSS version 21.0 software (IBM, Tokyo, Japan), with the level of statistical significance set at p <0.05.

## Abbreviations

ANCOVA: Analysis of covariance
ANOVA: Analysis of variance
ASIS: Anterior inferior iliac spine
EMG: Electromyography
IL: Iliopsoas
MRI: Magnetic resonance imaging
MVIC: Maximum voluntary isometric contraction
RF: Rectus femoris
RMS: Root mean square
SA: Sartorius
sEMG: Surface electromyography
TFL: Tensor fasciae latae.
